# Risk factors and metabolomics of mild cognitive impairment in type 2 diabetes mellitus

**DOI:** 10.3389/fmolb.2024.1341290

**Published:** 2024-04-18

**Authors:** Tao Luo, Xiao Jiang, Ning Xu, Xinyu Zhao, Xingjie Xie, Xiuwen Xia, XiaoLong Bian, Haixia Liu

**Affiliations:** ^1^ Endocrinology and Metabolism Department, The Second Affiliated Hospital of Dalian Medical University, Dalian, China; ^2^ Endocrinology Department, The Second Hospital of Chao Yang, Chaoyang, China; ^3^ The Second Clinical College, Dalian Medical University, Dalian, China

**Keywords:** type 2 diabetes mellitus, mild cognitive impairment, risk factors, metabolomics, LPC

## Abstract

**Objective:** This study aimed to explore the risk factors, metabolic characteristics, and potential biomarkers of mild cognitive impairment in type 2 diabetes mellitus (T2DM-MCI) and to provide potential evidence for the diagnosis, prevention, and treatment of mild cognitive impairment (MCI) in patients with type 2 diabetes mellitus (T2DM).

**Methods:** A total of 103 patients with T2DM were recruited from the Endocrinology Department of The Second Affiliated Hospital of Dalian Medical University for inclusion in the study. The Montreal Cognitive Assessment (MoCA) was utilized to evaluate the cognitive functioning of all patients. Among them, 50 patients were categorized into the T2DM-MCI group (MoCA score < 26 points), while 53 subjects were classified into the T2DM without cognitive impairment (T2DM-NCI) group (MoCA score ≥ 26 points). Serum samples were collected from the subjects, and metabolomics profiling data were generated by Ultra-high performance liquid chromatography-mass spectrometry (UHPLC-MS). These groups were analyzed to investigate the differences in expression of small molecule metabolites, metabolic pathways, and potential specific biomarkers.

**Results:** Comparison between the T2DM-MCI group and T2DM-NCI group revealed significant differences in years of education, history of insulin application, insulin resistance index, insulin-like growth factor-binding protein-3 (IGFBP-3), and creatinine levels. Further binary logistic regression analysis of the variables indicated that low educational level and low serum IGFBP-3 were independent risk factor for T2DM-MCI. Metabolomics analysis revealed that differential expression of 10 metabolites between the T2DM-MCI group and T2DM-NCI group (*p* < 0.05 and FDR<0.05, VIP>1.5). Kyoto Encyclopedia of Genes and Genomes (KEGG) enrichment pathway analysis revealed that fatty acid degradation was the most significant pathway. Receiver operating characteristic (ROC) analysis shows that lysophosphatidylcholine (LPC) 18:0 exhibited greater diagnostic efficiency.

**Conclusion:** This study revealed that a shorter duration of education and lower serum IGFBP-3 levels are independent risk factors for T2DM-MCI. Serum metabolites were found to be altered in both T2DM-MCI and T2DM-NCI groups. T2DM patients with or without MCI can be distinguished by LPC 18:0. Abnormal lipid metabolism plays a significant role in the development of MCI in T2DM patients.

## 1 Introduction

Type 2 Diabetes Mellitus (T2DM) is a prevalent subtype of diabetes mellitus (Srikanth et al., 2020) characterized by chronic hyperglycemia resulting from reduced insulin sensitivity in individuals. The International Diabetes Federation (IDF) estimated that 537 million adults aged 20–79 years had diabetes in 2021 (Sun et al., 2022). T2DM induces damage and dysfunction in a majority of tissues and organs (Harding et al., 2019), leading to severe complications such as diabetic ketoacidosis (DKA), hyperosmolar hyperglycaemic state (HHS), and MCI (Umpierrez and Korytkowski, 2016).

Cognitive function refers to the brain’s ability to receive, process, and transform external information. T2DM is a risk factor for cognitive dysfunction, which is now acknowledged as a complication of T2DM (Simó et al., 2017). Patients suffering from T2DM combined with cognitive impairment are less capable of managing their diabetes, leading to a vicious cycle between T2DM and cognitive dysfunction. This cycle substantially increases the health and financial burden on patients with T2DM who also experience cognitive impairment. However, the risk factors and specific mechanisms underlying cognitive impairment in T2DM patients have not yet been fully elucidated, and there is no uniform standard for the diagnosis and treatment of cognitive impairment in this population. Therefore, it is imperative to identify the controllable risk factors and pathogenesis of cognitive impairment in T2DM patients and to explore the therapeutic targets for the prevention and treatment of cognitive impairment in T2DM patients population in the future.

Metabolomics, characterized by its high sensitivity, throughput, and accuracy, is a research technique finding extensive applications in the medical field, particularly in the study of metabolic diseases. In clinical research, metabolomic changes are assessed, and biomarkers of disease are explored through metabolomics. Several studies based on metabolomic analysis techniques, utilizing various samples of small molecule metabolites from diabetic patients or animal models, have revealed metabolic abnormalities in individuals or animals with cognitive impairment compared to those with normal cognition (Song et al., 2017; Zhao et al., 2018; Sun et al., 2020; Chen et al., 2021). However, research involving non-targeted metabolomics of T2DM-MCI remains relatively scarce. Ultra-high performance liquid chromatography-mass spectrometry (UHPLC-MS), a commonly employed metabolomics technique known for its high sensitivity and coverage of the metabolome, is widely utilized in the analysis of non-targeted metabolomics biomarkers of disease.

In our research, we analyzed the risk factors for T2DM-MCI and identified metabolomic indicators using UHPLC-MS. We compared the differences in metabolic components between T2DM-NCI and T2DM-MCI, analyzed the abnormal metabolic pathways in T2DM-MCI, identified characteristic biomarkers of T2DM-MCI, and provided new insights for effective interventional treatment of cognitive impairment in T2DM patients in the future.

## 2 Methods and materials

### 2.1 Sample

The research received approved from the Ethics Committee of The Second Affiliated Hospital of Dalian Medical University (Approval Number: 2021 NO. 121). A total of 113 patients with T2DM admitted to the department of Endocrinology at The Second Affiliated Hospital of Dalian Medical University were enrolled in the T2DM group. The cognitive function of all subjects was assessed using the MoCA scale (Supplementary Figure S1). Among them, 50 patients with MoCA scores < 26 were categorized into the T2DM-MCI group, while 53 patients with MoCA scores ≥ 26 were classified into the T2DM-NCI group.

### 2.2 Diagnostic criteria

#### 2.2.1 T2DM diagnosis criteria

According to the 2022 diagnostic criteria of the American Diabetes Association (ADA) (American Diabetes Association Professional Practice Committee, 2022), diabetes is diagnosed in individuals presenting typical hyperglycemic symptoms and meeting one of the following conditions: (a) Fasting Plasma Glucose (FPG) ≥ 7.0 mmol/L; (b) Oral Glucose Tolerance Test (OGTT) ≥ 11.1 mmol/L; (c) Glycosylated Hemoglobin (HbA1c) ≥ 6.5%; (d) Random blood glucose ≥11.1 mmol/L in patients exhibiting typical symptoms of hyperglycemia or hyperglycaemic crisis.

Meeting the aforementioned diagnosis and the criteria indicates the presence of T2DM, characterized by: (a) Adult-onset, insidious onset, and chronic course; (b) Absence of tendency to ketosis in a non-stressed state; (c) Efficacy of hypoglycemic drugs; (d) Negative insulin-related antibodies, low insulin secretion curve, and C-peptide (C-P) release curve or delayed peak.

#### 2.2.2 Diagnostic criteria for MCI

The criteria for diagnosing MCI follow the guidelines established by the National Institute on Aging-Alzheimer’s Association (NIA-AA) (Albert et al., 2011), including: (a) Cognitive deficits reported by the patient, family member, or physician; (b) Objective evidence of impairment in one or more cognitive domains. In this study, cognitive function was assessed using the MoCA scale, with a MoCA score of ≥26 considered as NCI and a score of <26 indicating MCI. If the individual has 12 years of education or less, 1 point will be added to their MoCA score. (c) Maintaining independent living ability; (d) Absence of dementia.

#### 2.2.3 Diagnostic standards for hypoglycemia in diabetes

Hypoglycemia is a common complication in individuals with diabetes. The workgroup of the ADA proposed diabetic hypoglycaemia for clinical trials as follows: In numerical terms, a blood glucose value of 3.9 mmol/L (70 mg/dL) or lower with associated symptoms is usually considered a hypoglycemic condition (Workgroup on Hypoglycemia and American Diabetes Association, 2005).

### 2.3 Inclusion and exclusion criteria

#### 2.3.1 Inclusion criteria

The T2DM group includes patients who meet the following criteria: (a) Diagnosis of T2DM for more than 1 year; (b) No significant speech, visual or hearing impairment; (c) Ability to read and write independently.

#### 2.3.2 Exclusion criteria

All individuals meeting any of the following criteria will be excluded: (a) Traumatic brain injury, intracranial occupancy, cerebrovascular disease, epilepsy, anxiety, depression, dementia, and other psychiatric and neurological disorders; (b) Medications with cognitive impairment adverse effects; (c) History of cognitive impairment predating the diagnosis of T2DM; (d) Acute diabetic complications; (e) Inflammatory diseases, autoimmune diseases, hematological diseases, thyroid diseases, malignancies, cardiac, respiratory, hepatic, and renal failure, among others; (f) Chronic excessive alcohol consumption (more than 60 g per day); (g) Receipt of intravenous anesthesia within the past month.

### 2.4 Pre-treatment of serum samples

The patients’ serum was collected and analyzed using the UHPLC-MS technique. A total of 400 μL of methanol extract containing the internal standard (refer to Supplementary Table S1 for details on the internal standard content) was thoroughly mixed with 100 μL of serum sample. The mixture was then vortexed, centrifuged and divided into two separate portions of 180 μL each for analysis in positive and negative ion mode. Prior to analysis, the samples were re-solubilized with 50 μL of 25% acetonitrile. Blank samples were used to equilibrate the system before analyzing the actual samples. Furthermore, during the analysis, one quality control (QC) sample was injected after every 10 injections of the actual sample to monitor the stability of the sample pretreatment and instrument operation.

### 2.5 UHPLC-MS full component data acquisition method

A Waters BEH C8 column (size: 50 mm × 2.1 mm, 1.7 μm) (Waters, Milford, MA) was utilized for positive ion mode (ESI+) separation, employing a mobile phase consisting of water with 0.1% formic acid (A) and acetonitrile with 0.1% formic acid (B). The gradient began at 5% B and was maintained for 0.5 min, then increased to 40% B over 1.5 min, followed by a linear increase to 100% B over 6 min, which was held for 2 min before returning to the initial gradient of 5% B at 10.1 min and equilibrating for 2 min. An ACQUITY UPLC HSS T3 column (size: 50 mm × 2.1 mm, 1.8 μm) (Waters, Milford, MA) was utilized for negative ion mode (ESI-) separation. The mobile phases consisted of water spiked with 6.5 mM NH4HCO3 (A) and 95% methanol and 6.5 mM NH4HCO3 aqueous solution (B). The gradient commenced at 2% B and was maintained for 0.5 min, then increased to 40% B over 2 min, followed by a linear increase to 100% B over 6 min and held for 2 min before reverting to the initial gradient of 2% B at 10.1 min and equilibrating for 1.9 min. In both ion modes, the column temperature was set at 60°C and the elution flow rate was 0.4 mL/min.

The capillary temperature was set to 300°C, with auxiliary heating gas temperature at 350°C. Sheath and auxiliary gas flow rates were maintained at 45 and 10 (arbitrary units), respectively, while the full scan resolution was set to 7e4. For the positive ion mode, the m/z scan range was 80–1,200 Da, with a spray voltage of 3.50 kV. In the negative ion mode, the m/z scan range was 80–1,200 Da, with a spray voltage of 3.00 kV.

### 2.6 Statistical analysis

The normally distributed data from clinical parameters of the T2DM-MCI and T2DM-NCI groups were expressed as mean ± standard deviation. Variables with skewed distributions were described with 95% confidence intervals. The differences among the two groups were analyzed using *t*-tests or non-parametric tests.

All statistical analyses were performed using the Statistical Package for the Social Sciences (SPSS) version 25.0 (SPSS, Chicago, IL, United States). Results were considered statistically significant with two-tailed analyses at *p* < 0.05. Partial Least Squares Discriminant Analysis (PLS-DA) and Orthogonal Projections to Latent Structures Discriminant Analysis (OPLS-DA) were conducted using SIMCA version 14.1 (Umetrics AB, Umea, Sweden). Serum metabolites with a Variable Important in Projection (VIP) value greater than 1.5 in the OPLS-DA model were assessed for statistical significance using either a *t*-test or nonparametric test.

The data were normalized using MetaboAnalyst 5.0 (https://www.metaboanalyst.ca/) to reduce systematic bias and improve consistency. Features with >25% missing values were removed, and the remaining missing values were replaced by the mean in the original data. The data were then normalized by sum, mean-centered, and divided by the square root of the standard deviation of each variable. Enrichment analysis and metabolite heatmap generation were conducted using MetaboAnalyst. Correlation analysis was performed by Origin 2021. ROC curves were generated using SPSS to assess the diagnostic efficiency of differential metabolites. Metabolite point plotting was performed using Graph Prism 9.0.

## 3 Results

### 3.1 Risk factor analysis

#### 3.1.1 Baseline parameters

The clinical baseline parameters of the T2DM-MCI and the T2DM-NCI groups are shown in Table 1. In comparison to the T2DM-NCI group, the T2DM-MCI group exhibited a statistically significant difference in years of education (12.00 vs. 15.00 years) and history of insulin application (60.0% vs. 35.8%) (*p* < 0.05), while other parameters did not demonstrate significance (*p* > 0.05).

**TABLE 1:** Comparison of clinical parameters between the T2DM-MCI and the T2DM-NCI group.

Parameters	T2DM-MCI (*n* = 50)	T2DM-NCI (=53)	χ^2^/t/Z	*p*
Sex, M/F, n	23/27	33/20	2.743	0.098
Age, y	60.62 ± 5.696	58.60 ± 6.128	−1.731	0.087
Years of education, y	12.00 (9.00, 12.00)	15.00 (12.00, 16.00)	−4.515	<0.001*
T2DM, y	10.00 (5.75, 18.00)	10.00 (3.50, 16.00)	−1.366	0.172
Smoking, yes/no	11/39	15/38	0.541	0.462
Alcohol consumption, yes/no	6/44	6/47	0.012	0.914
Hypertension, yes/no	27/23	34/19	1.098	0.295
SBP, mmHg	143.00 (133.75, 154.50)	137.00 (125.50, 149.00)	−1.756	0.079
DBP, mmHg	81.94 ± 9.342	81.74 ± 12.312	−0.094	0.925
Diabetic peripheral neuropathy, yes/no	29/21	32/21	0.06	0.806
Diabetic macroangiopathy, yes/no	44/6	43/10	0.925	0.336
Diabetic nephropathy, yes/no	15/35	13/40	0.389	0.533
Diabetic retinopathy, yes/no	7/43	6/47	0.167	0.682
Metformin, yes/no	29/21	31/22	0.003	0.96
DPP-4 inhibitor, yes/no	5/45	7/46	0.257	0.612
GLP-1RA, yes/no	3/47	2/51	0.004	0.947
Insulin application, yes/no	30/20	19/34	6.017	0.014*
Hypoglycaemia, yes/no	23/27	16/37	2.734	0.098
Waist circumference, cm	93.00 (87.00, 101.00)	94.00 (90.00, 100.00)	−0.007	0.995
Hip circumference, cm	100.50 (95.00, 105.00)	98.00 (94.00, 103.00)	−1.051	0.293
BMI, kg/m^2^	25.27 (23.28, 26.68)	25.10 (23.03, 26.45)	−0.792	0.428

*Represent *p* < 0.05.

Clinical laboratory findings revealed no significant differences in HbA1c, FPG, insulin, C-P, Homeostatic Model Assessment of β (HOMA-β), insulin-like growth factor 1 (IGF-1), total cholesterol (TC), triglyceride (TG), high-density lipoprotein cholesterol (HDL-C), low-density lipoprotein cholesterol (LDL-C), Apolipoprotein (Apo) AⅠ, ApoB, Alanine Aminotransferase (ALT), Aspartate Aminotransferase (AST), Albumin (Alb), Interleukin (IL)-1β, IL-2R, IL-6, IL-8, IL-10, TNF-α, and Tumor Necrosis Factor-alpha (TNF-α) and Urinary Albumin (U-ALB)/Creatinine (Cr) between the T2DM-MCI and T2DM-NCI groups (*p* > 0.05). However, IGFBP-3 and Cr levels were notably lower, and HOMA-IR (Homeostatic Model Assessment of Insulin Resistance) was significantly higher in T2DM-MCI patients compared to T2DM-NCI subjects (*p* < 0.05) (Table 2).

**TABLE 2:** Comparison of clinical laboratory parameters between the T2DM-MCI and the T2DM-NCI group.

Parameters	T2DM-MCI (*n* = 50)	T2DM-NCI (*n* = 53)	χ^2^/t/Z	*p*
HbA1c, %	8.42 ± 1.57	8.12 ± 2.02	−0.818	0.415
FPG, mmol/L	8.18 ± 2.49	7.60 ± 2.11	−1.276	0.205
1 h PG, mmol/L	14.34 ± 3.92	13.93 ± 3.43	−0.569	0.571
2 h PG, mmol/L	15.90 (12.02, 18.89)	15.48 (12.32, 18.15)	−0.231	0.817
Ins, mU/L	11.11 (6.68, 20.83)	9.52 (5.53, 13.89)	−1.577	0.115
1 h Ins, mU/L	36.20 (21.20, 70.31)	25.83 (16.71, 50.36)	−1.775	0.076
2 h Ins, mU/L	40.69 (20.17, 62.56)	31.90 (17.42, 58.97)	−1.247	0.212
C-P, ng/mL	0.97 (0.56, 1.35)	1.17 (0.77, 1.79)	−1.835	0.067
1 h C-P, ng/mL	2.08 (1.26, 3.20)	2.39 (1.46, 3.44)	−1.095	0.273
2 h C-P, ng/mL	2.89 (1.57, 4.08)	3.58 (2.13, 5.10)	−1.755	0.079
HOMA-IR	4.68 (2.43, 6.98)	3.08 (1.68, 4.84)	−2.006	0.045*
HOMA-β	51.56 (28.94, 160.24)	54.68 (27.13, 101.00)	−0.468	0.639
IGF-1, ng/mL	145.97 ± 51.66	152.83 ± 37.38	0.776	0.44
IGFBP-3, ug/mL	4.10 (3.38, 4.66)	4.67 (4.15, 5.56)	−3.408	0.001*
IGF-1/IGFBP-3, ng/ng	0.03 (0.03, 0.04)	0.03 (0.03, 0.04)	−1.168	0.243
TC, mmol/L	4.85 ± 1.06	4.95 ± 1.18	0.422	0.674
TG, mmol/L	1.40 (0.97, 1.96)	1.60 (1.15, 2.02)	−1.488	0.137
HDL-C, mmol/L	1.17 ± 0.28	1.13 ± 0.22	−0.818	0.415
LDL-C, mmol/L	2.83 ± 0.86	2.85 ± 0.86	0.137	0.891
ApoAⅠ, g/L	1.39 (1.28, 1.50)	1.43 (1.29, 1.55)	−0.805	0.421
ApoB, g/L	0.88 ± 0.22	0.91 ± 0.23	0.682	0.497
ALT, U/L	18.82 (13.18, 34.11)	17.87 (13.28, 25.62)	−0.442	0.658
AST, U/L	17.72 (14.24, 23.26)	17.98 (14.83, 21.50)	−0.244	0.807
Alb, g/L	39.46 (37.48, 43.74)	41.10 (39.43, 43.61)	−1.801	0.072
Cr, μmol/L	57.62 ± 14.04	64.29 ± 16.05	2.247	0.027*
IL-1β, pg/mL	5.00 (5.00, 7.52)	5.00 (5.00, 7.09)	−0.193	0.847
IL-2R, U/mL	376.50 (298.25, 520.25)	338.00 (284.50, 450.50)	−1.359	0.174
IL-6, pg/mL	2.87 (2.03, 5.01)	2.86 (2.00, 4.99)	−0.08	0.936
IL-8, pg/mL	70.75 (22.28, 202.00)	88.00 (38.09, 229.00)	−1.594	0.111
IL-10, pg/mL	5.00 (5.00, 5.00)	5.00 (5.00, 5.00)	−1.463	0.143
TNF-α, pg/mL	12.35 (7.93, 37.98)	21.30 (9.56, 57.50)	−1.488	0.137
U-ALB/Cr, mg/gcr	29.03 (16.83, 47.13)	22.95 (12.35, 42.84)	−1.491	0.136

*Represent *p* < 0.05.

#### 3.1.2 Independent risk factors

Multifactorial binary logistic regression analysis was used to evaluate independent risk factors in T2DM patients with or without MCI. Years of education, history of insulin application, HOMA-IR, IGFBP-3, and Cr (*p* < 0.05 in Analysis of Variance) were employed as independent variables. The results indicated that short education and low serum IGFBP-3 levels were independent risk factors for MCI in T2DM patients. Specifically, each year of education in T2DM patients increased the risk of MCI by 22.2%, and each unit decrease in serum IGFBP-3 increased the risk of MCI by 51.7% (Refer to Table 3 for details).

**TABLE 3:** Multifactor binary logistic regression analysis of T2DM and MCI.

Parameters	Regression coefficient	Standard error	Wald	*p*	Odds ratio	95% CI
Years of education	−0.251	0.075	11.04	0.001*	0.778	0.671–0.902
Insulin application	0.735	0.544	1.828	0.176	2.086	0.719–6.056
HOMA-IR	0.075	0.055	1.877	0.171	1.078	0.968–1.200
IGFBP-3	−0.728	0.256	8.114	0.004*	0.483	0.292–0.797
Cr	−0.019	0.019	1.058	0.304	0.981	0.946–1.017

*Represent *p* < 0.05.

### 3.2 Metabolomic analysis

#### 3.2.1 Characteristics of samples

A total of 103 patients’ sera were collected, from which 12 samples were excluded due to sub-optimal quality. Subsequently, 47 cases in the T2DM-MCI group and 44 cases in the T2DM-NCI group were utilized for the metabolomic data analysis. The study flowchart is depicted in Supplementary Figure S2, and detailed demographic characteristics of the participants are presented in Tables 4, 5. Both groups exhibited significance in years of education, history of insulin application, IGFBP-3, Cr, and MoCA Score (*p* < 0.05). Furthermore, no significant differences were observed for sex, age, Body Mass Index (BMI), FPG, HbA1c, TC, TG, HDL-C, LDL-C, HOMA-IR, HOMA-β, and IGF-1 between the T2DM-MCI and T2DM-NCI groups (*p* > 0.05).

**TABLE 4:** Comparison of clinical parameters between the T2DM-MCI and the T2DM-NCI group.

Parameters	T2DM-MCI (*n* = 47)	T2DM-NCI (*n* = 44)	χ^2^/t/Z	*p*
Sex, M/F, n	21/26	28/16	−1.519	0.070
Age, y	61.13 ± 5.46	59.25 ± 6.33	−4.200	0.132
Years of education, y	12 (9, 12)	15 (12, 16)	−1.189	<0.001*
T2DM, y	10 (6, 18)	10 (4.25, 16)	0.177	0.238
Smoking, yes/no	10/37	11/33	0.015	0.674
Alcohol consumption, yes/no	6/41	6/38	2.217	0.902
Hypertension, yes/no	27/20	29/15	−1.960	0.136
SBP, mmHg	146.85 ± 20.30	138.23 ± 21.68	−0.299	0.053
DBP, mmHg	81.96 ± 9.54	82.66 ± 12.53	0.002	0.766
Diabetic peripheral neuropathy, yes/no	28/19	26/18	0.0150	0.963
Diabetic macroangiopathy, yes/no	41/6	38/6	0.060	0.902
Diabetic nephropathy, yes/no	15/32	13/31	0.029	0.807
Diabetic retinopathy, yes/no	7/40	6/38	0.004	0.864
Metformin, yes/no	27/20	25/19	0.192	0.952
DPP-4 inhibitor, yes/no	5/42	6/38	0.914	0.661
GLP-1RA, yes/no	3/44	1/43	4.837	0.339
Insulin application, yes/no	29/18	17/27	2.327	0.028*
Hypoglycaemia, yes/no	20/27	12/32	−0.135	0.127
Waist circumference, cm	93 (88, 101)	94 (90, 100)	−0.649	0.892
Hip circumference, cm	100 (95, 105)	100 (94.25, 103)	−0.560	0.516
BMI, kg/m2	25.24 (23.23, 26.57)	25.18 (23.01, 26.46)	−1.519	0.576

*Represent *p* < 0.05.

**TABLE 5:** Comparison of clinical laboratory parameters between the T2DM-MCI and the T2DM-NCI group.

Parameters	T2DM-MCI (*n* = 47)	T2DM-NCI (*n* = 44)	χ^2^/t/Z	*p*
HbA1c, %	8.36 ± 1.52	8.16 ± 2.08	−0.54	0.591
FPG, mmol/L	8.08 ± 2.47	7.87 ± 2.18	−0.434	0.665
1 h PG, mmol/L	14.23 ± 3.97	14.05 ± 3.37	−0.241	0.810
2 h PG, mmol/L	15.82 (11.18, 18.62)	15.57 (12.40, 17.90)	−0.016	0.987
Ins, mU/L	11.63 (6.68, 21.31)	9.76 (4.48, 15.35)	−1.636	0.102
1 h Ins, mU/L	36.43 (22.70, 74.91)	26.84 (18.20, 51.560)	−1.731	0.083
2 h Ins, mU/L	41.21 (20.98, 61.92)	32.01 (16.01, 59.56)	−1.326	0.185
C-P, ng/mL	0.98 (0.52, 1.35)	1.17 (0.76, 1.89)	−1.902	0.057
1 h C-P, ng/mL	2.10 (1.35, 3.18)	2.75 (1.41, 3.95)	−1.243	0.214
2 h C-P, ng/mL	2.89 (1.56, 4.07)	3.79 (1.86, 5.38)	−1.739	0.082
HOMA-IR	4.64 (2.48, 6.94)	3.13 (1.66, 5.16)	−1.708	0.088
HOMA-β	56.45 (27.86, 162.60)	53.54 (24.38, 95.42)	−1.088	0.277
IGF-1, ng/mL	148.57 ± 51.43	153.34 ± 37.93	0.501	0.618
IGFBP-3, ug/mL	4.09 (3.35, 4.67)	4.67 (4.12, 5.51)	−3.264	0.001*
IGF-1/IGFBP-3, ng/ng	0.03 (0.03, 0.04)	0.03 (0.03, 0.04)	−1.406	0.160
TC, mmol/L	4.79 ± 1.02	4.84 ± 1.08	0.229	0.819
TG, mmol/L	1.39 (0.93, 1.94)	1.46 (1.12, 1.98)	−1.017	0.309
HDL-C, mmol/L	1.18 ± 0.28	1.16 ± 0.22	−0.454	0.651
LDL-C, mmol/L	2.76 ± 0.83	2.81 ± 0.89	0.284	0.777
ApoAⅠ, g/L	1.43 (1.29, 1.55)	1.40 (1.28, 1.50)	−0.584	0.559
ApoB, g/L	0.87 ± 0.21	0.91 ± 0.23	1.004	0.318
ALT, U/L	19.05 (13.22, 34.59)	17.78 (12.90, 25.69)	−0.786	0.432
AST, U/L	18.32 (14.98, 23.32)	18.07 (14.64, 21.20)	−0.627	0.530
Alb, g/L	39.61 (37.49, 43.93)	41.66 (39.48, 43.75)	−1.656	0.098
Cr, μmol/L	56.47 (46.61, 67.74)	64.12 (51.51, 73.52)	2.303	0.021*
IL-1β, pg/mL	5.00 (5.00, 7.47)	5.00 (5.00, 6.50)	−0.536	0.592
IL-2R, U/mL	377.00 (302.00, 517.00)	360.50 (283.75, 462.50)	−1.152	0.249
IL-6, pg/mL	2.83 (2.02, 4.90)	2.79 (2.00, 4.74)	−0.128	0.898
IL-8, pg/mL	67.90 (21.90, 170.00)	87.73 (34.33, 227.50)	−1.473	0.141
IL-10, pg/mL	5.00 (5.00, 5.00)	5.00 (5.00, 5.00)	−1.376	0.169
TNF-α, pg/mL	11.80 (7.93, 34.95)	19.05 (9.71, 51.40)	−1.441	0.149
U-ALB/Cr, mg/gcr	31.51 (16.43, 51.45)	23.29 (11.70, 42.23)	−1.501	0.133
MoCA Score	23.00 (21.00, 25.00)	28.00 (27.00, 29.00)	−8.267	0.001*

*Represent *p* < 0.05.

#### 3.2.2 Differences in serum metabolic profiling

**FIGURE 1:**
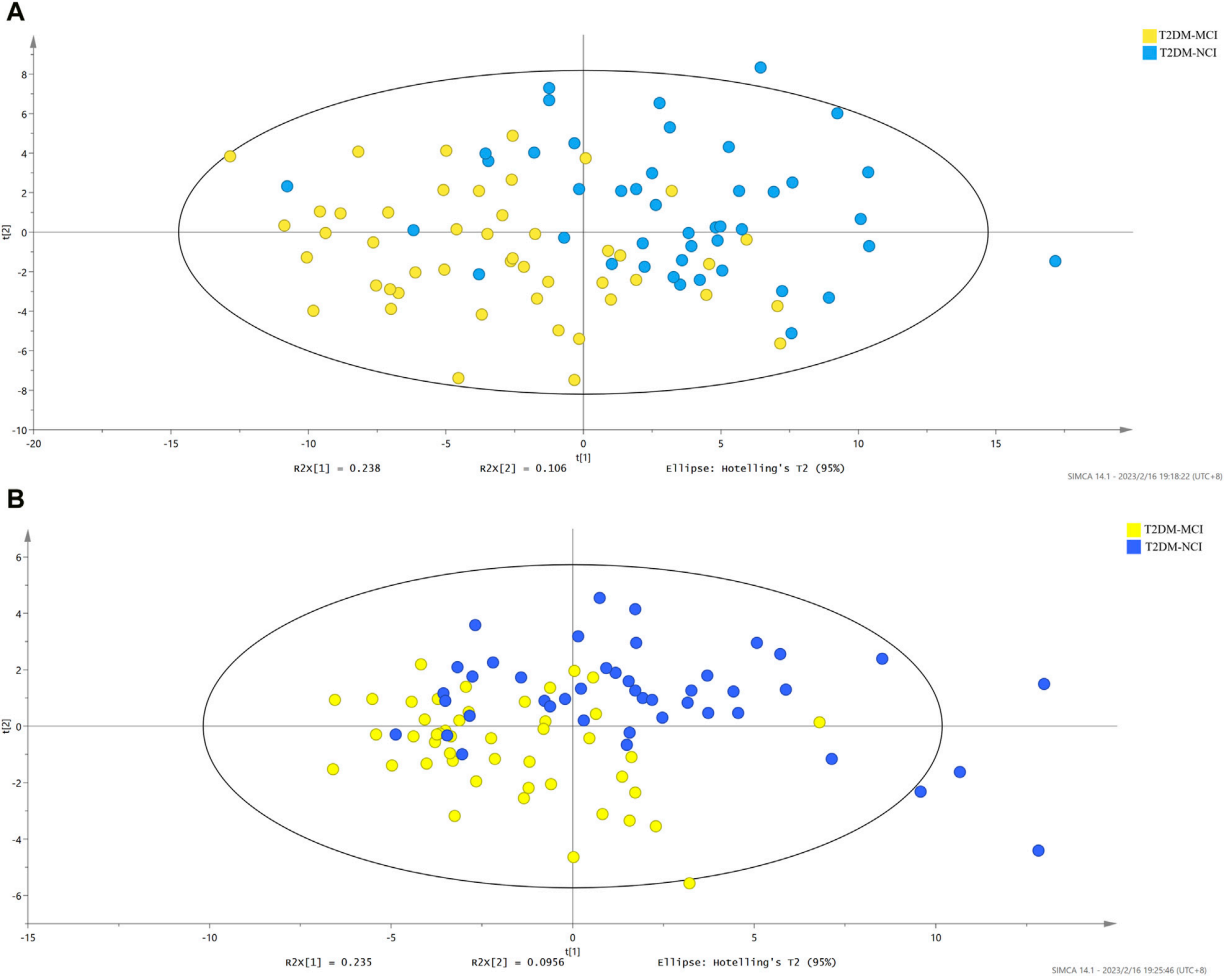
**(A)** PLS-DA score plot of the T2DM-MCI group vs. the T2DM-NCI group in ESI+ (R2X = 0.343, R2Y = 0.436, Q2 = 0.238). **(B)** PLS-DA score plot of the T2DM-MCI group vs. the T2DM-NCI group in ESI- (R2X = 0.33, R2Y = 0.421, Q2 = 0.269).

Partial Least Squares-Discriminant Analysis (PLS-DA) was performed on the metabolomics data, revealing differences in metabolic profiles between the T2DM-MCI and T2DM-NCI groups, indicating aberrant metabolism in T2DM-MCI. The permutation test plots (Figures 1A, B) exhibited permutation test intercepts of (ESI+) R2X = 0.343, R2Y = 0.436, Q2 = 0.238, and (ESI−) R2X = 0.33, R2Y = 0.421, Q2 = 0.269 for the positive and negative ion models, respectively.

**FIGURE 2:**
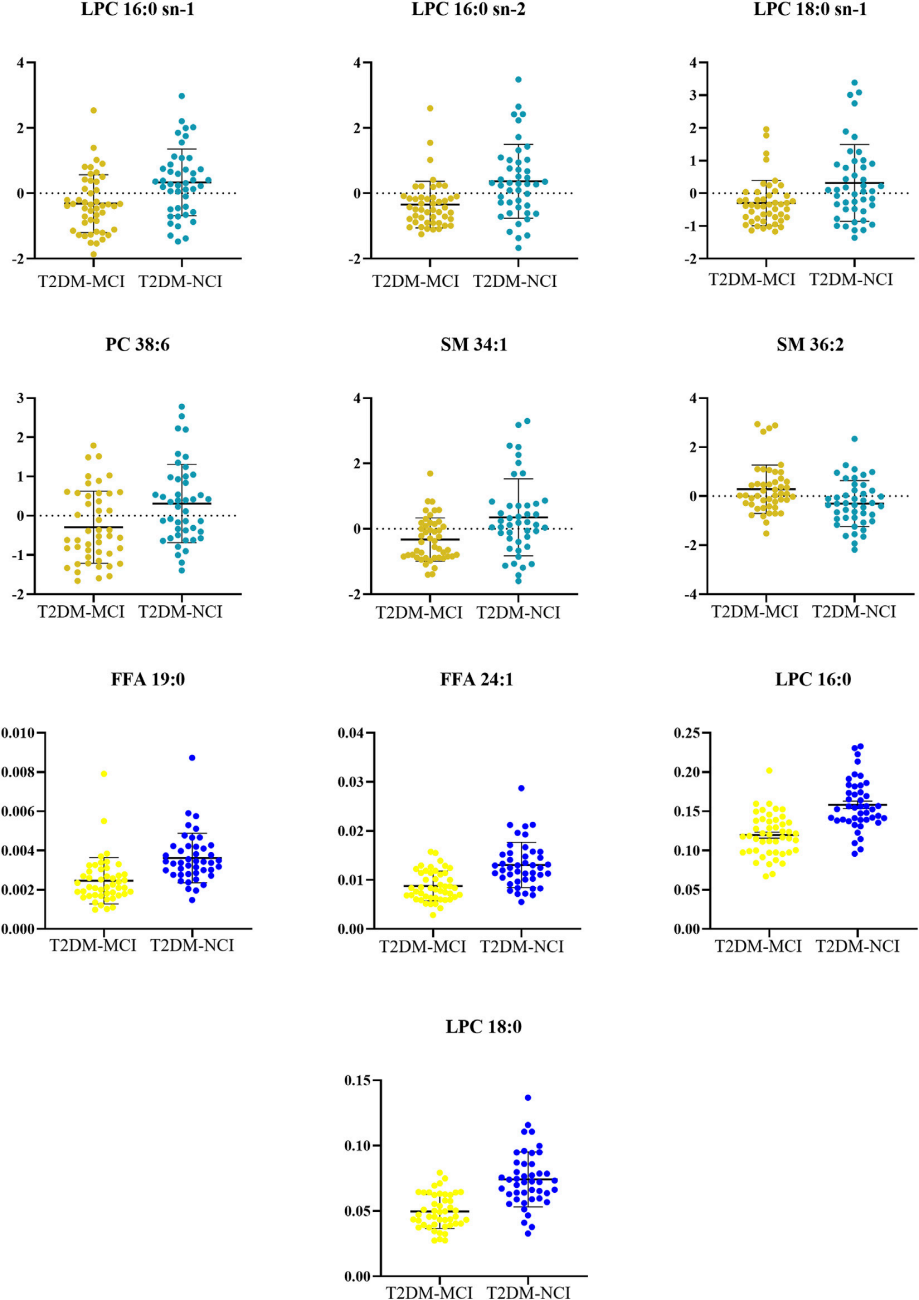
The metabolites from comparison between the T2DM-MCI and the T2DM-NCI group.

**FIGURE 3:**
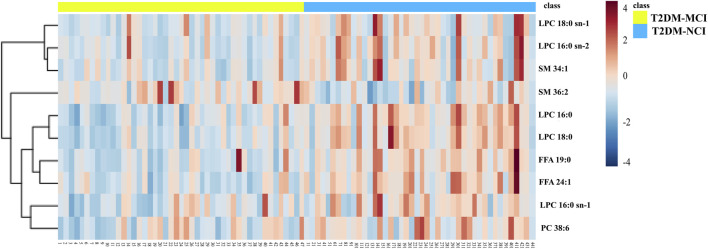
The heatmap metabolites from comparison between the T2DM-MCI and the T2DM-NCI group.

Between-group differences were assessed using independent samples *t*-tests or Mann-Whitney U tests. Based on the VIP and significant differences between the two groups, 10 metabolites were identified (*p* < 0.05, VIP > 1.5, and FDR < 0.05, as shown in Table 6). In the comparison between the T2DM-MCI and T2DM-NCI groups, lysophosphatidylcholine (LPC) 16:0 sn-1, LPC 16:0 sn-2, LPC 18:0 sn-1, phosphatidylcholine (PC) 38:6, sphingomyelin (SM) 34:1, free fatty acid (FFA) 19:0, FFA 24:1, LPC 16:0, and LPC 18:0 were higher in T2DM-NCI patients, while SM 36:2 was lower (Figure 2; Table 6). The heatmap of metabolites is displayed in Figure 3.

**TABLE 6:** Identified metabolites between the T2DM-MCI and the T2DM-NCI group.

Metabolites	Acquisition mode	Var ID (Primary)	T2DM-MCI (*n* = 47)	T2DM-NCI(*n* = 44)	*p*
LPC 16:0 sn-1	ESI+	5.28262	−0.376 (−1.109, 0.376)	0.296 (−0.480, 0.849)	0.003
LPC 16:0 sn-2	ESI+	1.97096	−0.489 (−0.882, −0.121)	0.294 (−0.415, 1.000)	<0.001*
LPC 18:0 sn-1	ESI+	3.93647	−0.382 (−0.769, −0.063)	0.0850 (−0.485, 0.900)	0.006
PC 38:6	ESI+	3.09121	−0.292 ± 0.918	0.312 ± 0.151	0.003
SM 34:1	ESI+	4.13457	−0.401 (−0.847, 0.147)	0.155 (−0.371, 0.753)	0.003
SM 36:2	ESI+	2.81402	0.030 (−0.335, 0.589)	−0.336 (−0.919, 0.233)	0.006
FFA 19:0	ESI-	1.5599	0.002 (0.002, 0.003)	0.003 (0.003, 0.004)	<0.001*
FFA 24:1	ESI-	1.66994	0.009 ± 0.003	0.013 ± 0.005	<0.001*
LPC 16:0	ESI-	2.2399	0.120 ± 0.027	0.158 ± 0.032	<0.001*
LPC 18:0	ESI-	2.51154	0.050 ± 0.013	0.074 ± 0.021	<0.001*

*Represent *p* < 0.05.

#### 3.2.3 ROC curve

**FIGURE 4:**
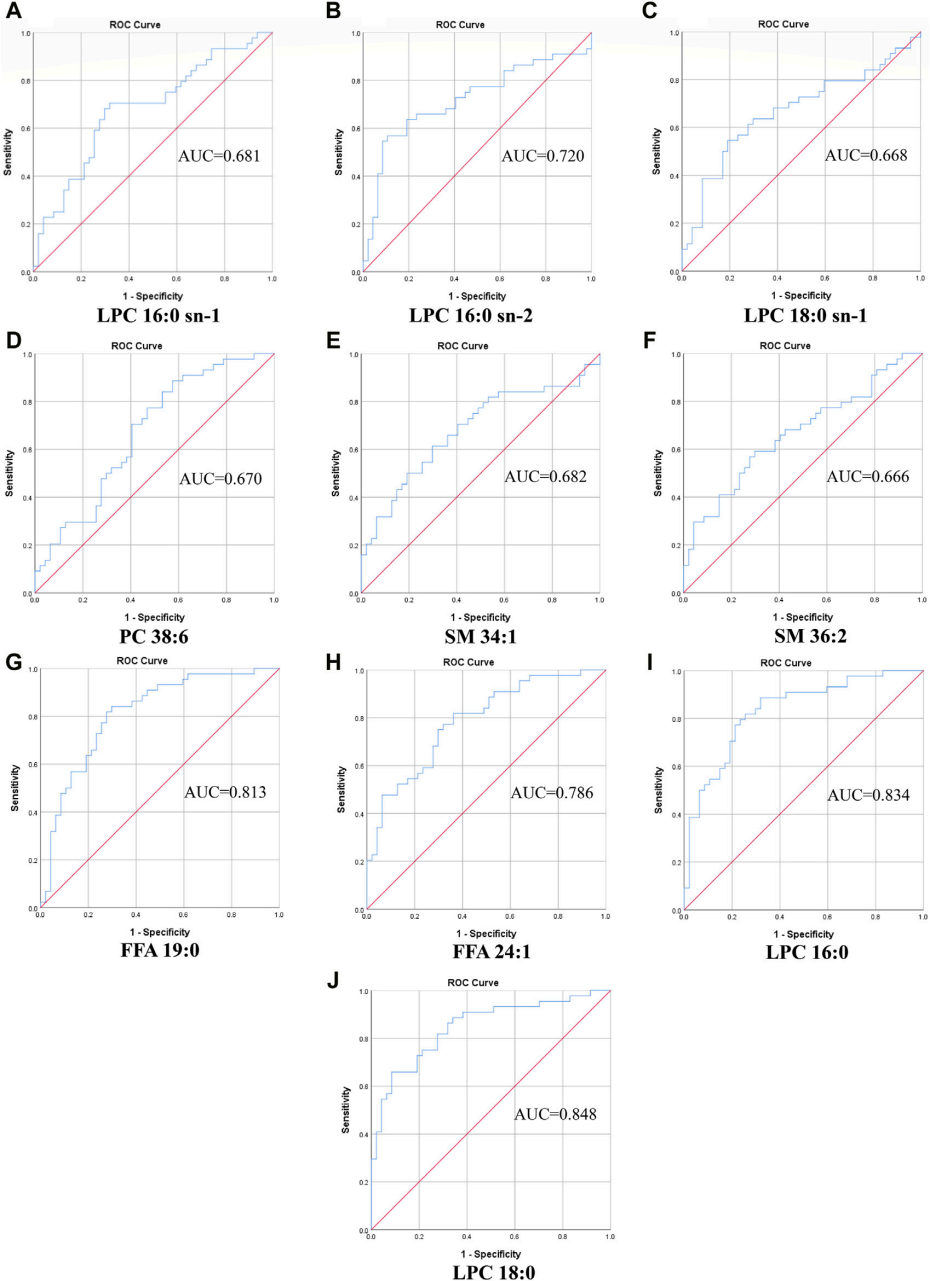
The ROC curve of selected metabolomics. **(A)** LPC 16:0 sn-1, AUC = 0.681. **(B)** LPC 16:0 sn-2, AUC = 0.720. **(C)** LPC 18:0 sn-1, AUC = 0.668. **(D)** PC 38:6, AUC = 0.670. **(E)** SM 34:1, AUC = 0.682. **(F)** SM 36:2, AUC = 0.666. **(G)** FFA 19:0, AUC = 0.813. **(H)** FFA 24:1, AUC = 0.786. **(I)** LPC16:0, AUC = 0.834. **(J)** LPC18:0, AUC = 0.848.

We calculated the area under the curve (AUC), sensitivity, and specificity of the metabolites screened as potential biomarkers, which showed that each metabolite had good predictability (AUC > 0.6, Figures 4A–I, Table 7). LPC18:0 was the most diagnostically efficient in distinguishing T2DM-MCI from T2DM-NCI (Figure 4J, AUC = 0.848, 95% CI: 0.767–0.928). Additionally, the AUC values for FFA 19:0 and LPC16:0 were also greater than 0.8 (Figures 4G, I).

**TABLE 7:** Receiver operating characteristic curve analysis of 10 metabolites.

Metabolites	AUC	Cut-off point	Sensitivity, %	Specificity, %	Maximum of youden index	*p*
LPC 16:0 sn-1	0.681	−0.147	0.705	0.681	0.386	0.003*
LPC 16:0 sn-2	0.720	0.237	0.568	0.894	0.462	<0.001*
LPC 18:0 sn-1	0.668	0.052	0.545	0.809	0.354	0.006*
PC 38:6	0.670	−0.657	0.886	0.426	0.312	0.005*
SM 34:1	0.682	0.018	0.614	0.702	0.316	0.003*
SM 36:2	0.666	−0.172	0.591	0.702	0.293	0.006*
FFA 19:0	0.813	0.003	0.841	0.702	0.543	<0.001*
FFA 24:1	0.786	0.009	0.818	0.638	0.456	<0.001*
LPC16:0	0.834	0.130	0.886	0.681	0.567	<0.001*
LPC18:0	0.848	0.065	0.659	0.915	0.574	<0.001*

AUC: area under the curve; Maximum of youden index = Sensitivity + specificity–1; *Represent *p* < 0.05.

#### 3.2.4 Enrichment analysis

**FIGURE 5:**
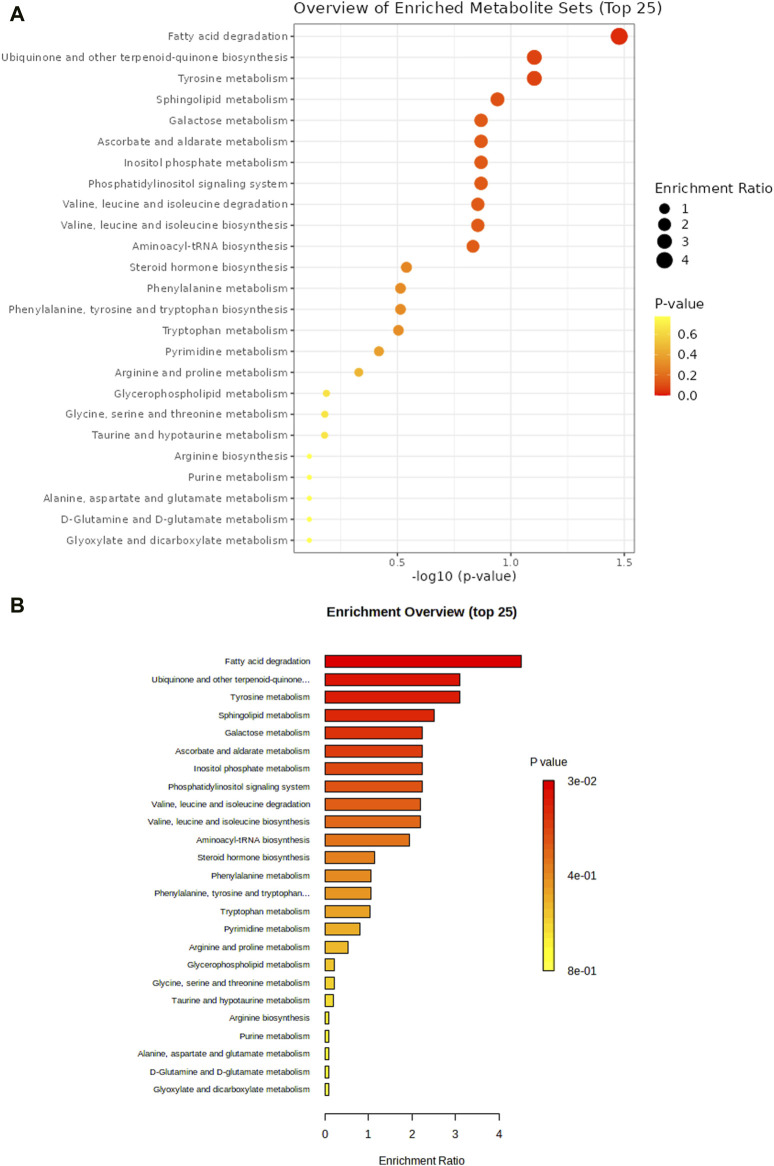
Enrichment analysis was performed to confirm the significantly changed metabolic pathway based on the Kyoto Encyclopedia of Genes and Genomes database through Metaboanalyst **(A,B)**.

Enrichment analysis was conducted to confirm the significantly altered metabolic pathways based on the Kyoto Encyclopedia of Genes and Genomes (KEGG) database using Metaboanalyst. Fatty acid degradation, ubiquinone and other terpenoid-quinone biosynthesis, and tyrosine metabolism had a significant impact on T2DM-MCI (Figures 5A, B).

## 4 Disscussion

T2DM and MCI are highly prevalent among middle-aged and elderly individuals, with a global increase in incidence attributed to population aging. Accumulating epidemiological evidence supports the notion that T2DM serves as a risk factor for cognitive impairment (Xue et al., 2019; You et al., 2021). Consequently, our study focused on analyzing the risk factors associated with T2DM-MCI, utilizing metabolomic analysis to investigate aberrant metabolic profiles. This approach facilitated the identification of potential specific biomarkers, offering valuable insights for the prevention, early diagnosis, and interventional treatment of T2DM-MCI.

Our research indicated that a shorter duration of education served as an independent risk factor for T2DM-MCI, consistent with previous findings (Exalto et al., 2013; Sun et al., 2020; Suain Bon et al., 2021). Secondary and higher education were associated with lower odds of cognitive impairment compared to those with no formal education or primary education only (Suain Bon et al., 2021). Domestic studies further demonstrated that receiving more than 12 years of education served as an independent protective factor (Xu et al., 2021). Sun et al. (2020) observed that individuals with higher levels of education and those engaged in intellectually stimulating occupations tend to exhibit a higher density of synapses in the cerebral cortex, subsequently enhancing the brain’s storage capacity and delaying dementia symptoms. Consequently, both T2DM patients and those without the condition should aim to improve their educational attainment and engage in brain-stimulating activities to reduce the risk of cognitive impairment.

Our findings suggest that decreased serum levels of IGFBP-3 serve as an independent risk factor for T2DM-MCI, consistent with the observations of Wennberg et al., who reported a positive association between higher IGFBP-3 levels and cognition among female subjects (Wennberg et al., 2018). Furthermore, Duron et al. (2012)’s study showed a significant increase in IGFBP-3 levels in the T2DM-MCI group compared to the control group, suggesting that IGFBP-3 also contributes to preserving cognitive function.

In our study, a statistically significant difference was observed in the history of insulin application between the T2DM-MCI group and the T2DM-NCI group. The potential mechanism involves the Insulin-degrading Enzyme (IDE), which degrades both insulin and amyloid-β (Aβ). Insulin competes with Aβ for IDE, leading to reduced degradation of Aβ and an increased risk of cognitive impairment due to Aβ deposition in the brain (Qiu and Folstein, 2006). Additionally, some scholars have suggested that T2DM patients receiving insulin treatment may experience a longer disease duration, increased complications, poorer islet function, and hypoglycemia, all of which can contribute to brain impairment.

Previous studies have reported that hypoglycemic episodes may increase the risk of cognitive impairment (Ye et al., 2024). In our study, although the frequency of hypoglycemic episodes in both groups of patients did not reach statistical significance, the T2DM-MCI group showed a higher occurrence of hypoglycemic episodes compared to the T2DM-NCI group.

Our findings suggest a link between insulin resistance (IR) and cognitive decline among patients with type 2 diabetes mellitus (T2DM). IR is characterized by decreased insulin sensitivity in target organs or tissues, leading to suboptimal biological effects of insulin secretion. This condition triggers compensatory elevations in peripheral insulin levels to sustain normal insulin function. IR has been recognized as a risk factor for mild cognitive impairment (MCI) in patients with T2DM (Kong et al., 2018; Pal et al., 2018). The pathogenesis of cognitive impairment associated with IR is complex and may encompass hyperinsulinemia, resistance to IGF-1, IR deposition, tau protein phosphorylation, inflammatory response, and oxidative stress. These processes lead to pathological changes closely linked to Alzheimer’s disease (AD), such as amyloid plaque formation, neuronal degeneration, and cognitive decline (Takahashi et al., 2017; Kellar and Craft, 2020). IR can be effectively managed in patients with T2DM through dietary and exercise interventions, which may improve IR and mitigate the risk of IR-related cognitive impairment.

Our findings demonstrated that the serum creatinine level in the T2DM-MCI group was significantly lower (57.62 ± 14.04 umol/L) compared to the T2DM-NCI group (64.29 ± 16.05 umol/L). Consistently, metabolomic analysis revealed lower creatinine levels in the T2DM-MCI group, indicating a potential association between decreased serum creatinine levels and cognitive impairment among T2DM patients. Serum creatinine primarily arises from muscle creatine and serves as a reliable marker of muscle mass in healthy individuals. Skeletal muscle, being a primary target tissue for insulin, exhibits a strong association with T2DM. Elevated blood glucose levels and peripheral nerve and vascular complications among T2DM patients are associated with decreased muscle mass, as reported in (Izzo et al., 2021). Evidence supports a correlation between decreased skeletal muscle mass and function, and impaired cognitive function, as cited in (Sui et al., 2020). Furthermore, additional evidence indicates that creatine supplementation may enhance cognitive function, as referenced in (Rawson and Venezia, 2011). Consequently, T2DM patients can potentially mitigate cognitive impairment by engaging in creatine supplementation and physical activity. Serum creatinine levels could potentially serve as a marker for identifying individuals at high risk of cognitive impairment among T2DM patients without kidney disease.

Besides the aforementioned factors, domestic scholars have identified various risk factors for T2DM-MCI, including age, diabetes duration, fasting blood glucose (FBG), HbA1c, low serum C-P levels, TC TG, LDL-C, waist circumference, and BMI. Additionally, moderate alcohol consumption and HDL-C levels serve as protective factors for T2DM-MCI, whereas diabetic macrovascular and microangiopathy are strongly associated with cognitive decline among patients, as documented in (Sun et al., 2020; Xia et al., 2020).

Previous studies have reported associations between cognitive decline and interleukin-1β (IL-1β), hypoglycemia, hypertension, elevated diabetic complications, smoking, and excessive alcohol consumption (Gorska-Ciebiada et al., 2016; Nilsson et al., 2019). Contrary to the aforementioned studies, our investigation did not detect any significant differences in age, disease duration, diabetic complications, smoking and alcohol consumption habits, hypertension, hypoglycemia, waist circumference, BMI, blood glucose levels, C-peptide concentrations, lipid profiles, or inflammatory markers. We hypothesize that these disparities may be attributed to variations in study design, sample size, assessment methodologies, and unique characteristics of T2DM.

We performed a serum metabolomic analysis on samples from T2DM-MCI patients and T2DM-NCI controls, identifying abnormalities in 10 metabolites between the T2DM-MCI and T2DM-NCI groups. Notably, decreased levels were observed in T2DM-MCI patients for LPC 16:0 sn-1, LPC 16:0 sn-2, LPC 18:0 sn-1, LPC 16:0, LPC 18:0, PC 38:6, SM 34:1, FFA 19:0, and FFA 24:1, whereas SM 36:2 levels were elevated. Additionally, LPC18:0 emerged as a potential biomarker for T2DM-MCI through ROC curve analysis.

Phospholipids play several crucial biological roles in the human body, and there is substantial evidence indicating that disorders of phospholipid metabolism are associated with cognitive impairment (Mapstone et al., 2014; Gorska-Ciebiada et al., 2016; Zhang et al., 2017; Nilsson et al., 2019; Xia et al., 2020). Phospholipids are classified into several categories based on their molecular structure, including phosphatidylcholine (PC), sphingomyelin (SM), and other phospholipids. These compounds serve as integral components of cell membranes in living organisms, playing a crucial role in maintaining membrane integrity and physiological functions. Previous research has reported abnormalities in membrane phospholipids in patients with Alzheimer’s disease (AD) (Nitsch et al., 1992). Mapstone et al. (2014) found significantly lower plasma phosphatidylcholine (PC) levels in subjects with cognitive impairment compared to controls, a finding that is consistent with our results. This could be attributed to the physiological function of phosphatidylcholine (PC), which encompasses regulating glucose and lipoprotein homeostasis, in addition to serving as a constituent part of cell membranes (Vance, 2008; Furse and de Kroon, 2015). In a prospective study involving 2,497 participants with a follow-up period exceeding 2 years, Ylilauri et al. (2019) found that higher dietary intake of phosphatidylcholine (PC) was associated with a lower risk of dementia and improved cognitive performance. Therefore, patients with type 2 diabetes mellitus and mild cognitive impairment (T2DM-MCI) may mitigate the progression of cognitive impairment by consuming foods rich in phosphatidylcholine (PC).

LPC levels are significantly reduced in schizophrenia patients compared to healthy controls, indicating a correlation between LPC levels and cognitive function (Orešič et al., 2012). Mapstone et al. (2014) reported a transition from normal cognition to MCI among subjects with depleted plasma LPC levels compared to controls with cognitive impairment. LPC serves as the primary carrier of docosahexaenoic acid (DHA) across the blood-brain barrier, supporting brain function through its multiple biological effects (Lagarde et al., 2001). LPC has also been implicated in demyelination, inflammatory responses, atherosclerosis, and other detrimental biological effects on cognitive function, contrary to previous evidence (Song et al., 2021).

We observed a downregulation of SM in the T2DM-MCI group in our study. Consistent with our findings, a study demonstrated significantly lower plasma SM levels in AD patients compared to controls (Han et al., 2011). Conversely, other studies have found that elevated SM levels are associated with the severity of AD pathology at autopsy and its progression in prodromal and preclinical stages (Varma et al., 2018). Mielke et al. (2010) conducted a study among elderly women, revealing that serum SM levels fluctuate depending on the timing of memory impairment episodes, with low levels associated with memory impairment and high levels predictive of future memory impairment up to 9 years later. This indicates that serum SM may serve as a reliable preclinical predictor or biomarker for memory impairment (Mielke et al., 2010). Further investigation into the relationship between SM and cognition across various cognitive function stages and different populations is essential.

The metabolism of fatty acids is significantly disrupted in T2DM-MCI. Fatty acids undergo catabolism for energy production through fatty acid β-oxidation. In our research, the serum levels of FFA were decreased in T2DM-MCI patients. Previous studies have consistently demonstrated lower FFA levels in AD patients compared to controls, encompassing various saturated and unsaturated fatty acids, such as FFA 16:0 and FFA 18:0. However, elevated levels of FFA 18:3 were observed, aligning with our results. It is important to note that the impact of different types of FFA on cognition may differ. Unsaturated fatty acids have been shown to preserve cell membrane fluidity, suppress inflammatory processes, enhance vascular endothelial cell function, inhibit platelet aggregation, and regulate lipid metabolism (Wiktorowska-Owczarek et al., 2015).

## 5 Conclusion

This study suggests that T2DM patients with low education level, history of insulin application, high insulin resistance, low serum IGFBP-3, and low creatinine values are more susceptible to MCI. Shorter duration of education and low serum IGFBP-3 levels are independent risk factors for T2DM-MCI. There are significant differences in serum metabolites between T2DM-MCI and T2DM-NCI. Abnormal lipid metabolism plays a important role in the development of cognitive impairment in T2DM patients. LPC 18:0 can effectively differentiate T2DM-MCI and T2DM-NCI. This study aims to identify characteristic biomarkers of MCI in patients with T2DM, thereby providing new insights for the effective intervention and treatment of cognitive impairment in this population in the future.

### 5.1 Limitations

Although this study reports novel findings, it has several limitations. Firstly, our study is a cross-sectional study with a small sample size and relatively limited results, which reduce the ability to detect risk factors for T2DM-MCI, and further validation is necessary. Secondly, predictive signatures may vary among different studies due to differences in the genetic and environmental background of the study population. Therefore, we plan to further expand the sample size in future studies and conduct additional research to validate the identified biomarkers.

## Figures and Tables

**Table:** 

**T2DM-MCI**	type 2 diabetes mellitus with mild cognitive impairment
**MCI**	mild cognitive impairment
**T2DM**	type 2 diabetes mellitus
**MoCA**	Montreal Cognitive Assessment
**T2DM-NCI**	T2DM without cognitive impairment
**UHPLC-MS**	Ultra-high performance liquid chromatography-mass spectrometry
**IGFBP-3**	insulin-like growth factor-binding protein-3
**IR**	Insulin Resistance
**KEGG**	Kyoto Encyclopedia of Genes and Genomes
**LPC**	lysophosphatidylcholine
**IDF**	International Diabetes Federation
**DKA**	diabetic ketoacidosis
**HHS**	Hyperosmolar Hyperglycaemic State
**ADA**	American Diabetes Association
**FPG**	Fasting Plasma Glucose
**OGTT**	Oral glucose tolerance test
**HbA1c**	Glycosylated Hemoglobin (HbA1c)
**NIA-AA**	National Institute onAging-Alzheimer’s Association
**QC**	quality control
**SPSS**	Statistical Package for the Social Sciences
**ESI+**	positive ion mode
**ESI-**	negative ion mode
**PLS-DA**	Partial Least Squares-Discriminant Analysis
**OPLS-DA**	Orthogonal Projections to Latent Structures Discriminant Analysis
**VIP**	Variable Important in Projection
**ROC**	Receiver operating characteristic
**C-P**	C-peptide
**HOMA-IR**	Homeostatic Model Assessment of Insulin Resistance
**HOMA-β**	Homeostatic Model Assessment of β
**IGF-1**	insulin-like growth factor 1
**TC**	total cholesterol
**TG**	triglyceride
**HDL-C**	high-density lipoprotein cholesterol
**LDL-C**	low-density lipoprotein cholesterol
**Apo**	Apolipoprotein
**ALT**	Alanine Aminotransferase
**AST**	Aspartate Aminotransferase
**Alb**	Albumin
**IL**	Interleukin
**TNF-α**	Tumor Necrosis Factor-alpha
**U-ALB**	Urinary Albumin
**Cr**	Creatinine
**PC**	phosphatidylcholine
**SM**	sphingomyelin
**FFA**	free fatty acid
**BMI**	Body Mass Index
**AUC**	area under the curve
**Aβ**	amyloid-β
**IDE**	Insulin-degrading Enzyme
**AD**	Alzheimer’s disease
**IL-1β**	interleukin-1β
**DHA**	docosahexaenoic acid

## Data Availability

The original contributions presented in the study are included in the article/Supplementary Material, further inquiries can be directed to the corresponding author.
