# Supplementary material for: Risk factors and metabolomics of mild cognitive impairment in type 2 diabetes mellitus
Source: Front Mol Biosci. 2024 Apr 18;11:1341290. doi: 10.3389/fmolb.2024.1341290 (PMC11063278; doi:10.3389/fmolb.2024.1341290)
Supplement: Supplementary file 1 [file Table1.docx]

**Table S1.**

**Internal standards for serum sample analysis**

| **Abbreviation** | **Ultimate concentration (ug/ml)** |
| --- | --- |
| Carnitine C2:0-d3 | 0.1 |
| Carnitine C10:0-d3 | 0.1 |
| Carnitine C16:0-d3 | 0.15 |
| LPC 19:0 | 0.75 |
| FFA C16:0-d3 | 2.5 |
| FFA C18:0-d3 | 2.5 |
| Trp-d5 | 4.25 |
| Phe-d5 | 3.6 |
| CA-d4 | 1.85 |
| CDCA-d4 | 1.5 |
