# Supplementary material for: Risk factors and metabolomics of mild cognitive impairment in type 2 diabetes mellitus
Source: Front Mol Biosci. 2024 Apr 18;11:1341290. doi: 10.3389/fmolb.2024.1341290 (PMC11063278; doi:10.3389/fmolb.2024.1341290)

Figure S1. Montreal Cognitive Assessment (MoCA)

MONTREAL COGNITIVE ASSESSMENT (MOCA)
Version 7.1 Original Version

NAME :
Education : Date of birth :
Sex : DATE :

		POINTS
**VISUOSPATIAL / EXECUTIVE**		

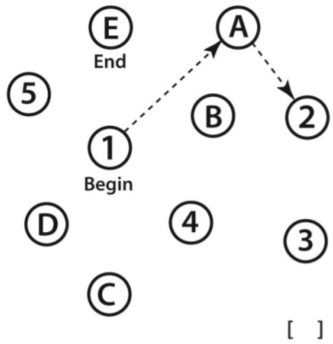

[ ]

Copy cube

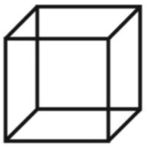

[ ]

Draw CLOCK (Ten past eleven)
(3 points)

[ ] Contour [ ] Numbers [ ] Hands

__/5

NAMING

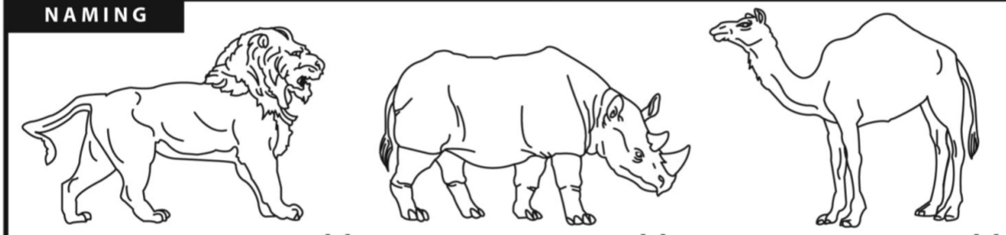

[ ] [ ] [ ]

__/3

MEMORY Read list of words, subject must repeat them. Do 2 trials, even if 1st trial is successful. Do a recall after 5 minutes.

	FACE	VELVET	CHURCH	DAISY	RED
1st trial					
2nd trial					

No points

ATTENTION Read list of digits (1 digit/ sec.).
Subject has to repeat them in the forward order [ ] 2 1 8 5 4
Subject has to repeat them in the backward order [ ] 7 4 2 __/2

Read list of letters. The subject must tap with his hand at each letter A. No points if ≥ 2 errors
[ ] F B A C M N A A J K L B A F A K D E A A A J A M O F A A B __/1

Serial 7 subtraction starting at 100 [ ] 93 [ ] 86 [ ] 79 [ ] 72 [ ] 65
4 or 5 correct subtractions: **3 pts**, 2 or 3 correct: **2 pts**, 1 correct: **1 pt**, 0 correct: **0 pt** __/3

LANGUAGE Repeat : I only know that John is the one to help today. [ ]
The cat always hid under the couch when dogs were in the room. [ ] __/2

Fluency / Name maximum number of words in one minute that begin with the letter F [ ] _____ (N ≥ 11 words) __/1

ABSTRACTION Similarity between e.g. banana - orange = fruit [ ] train – bicycle [ ] watch - ruler __/2

**DELAYED RECALL**	Has to recall words **WITH NO CUE**	FACE [ ]	VELVET [ ]	CHURCH [ ]	DAISY [ ]	RED [ ]	Points for UNCUED recall only	__/5
**Optional**	Category cue							
	Multiple choice cue							

ORIENTATION [ ] Date [ ] Month [ ] Year [ ] Day [ ] Place [ ] City __/6

© Z.Nasreddine MD **www.mocatest.org** Normal ≥26 / 30 TOTAL __/30
Add 1 point if ≤ 12 yr edu

Administered by: ________________

Figure S2. Flow Chart

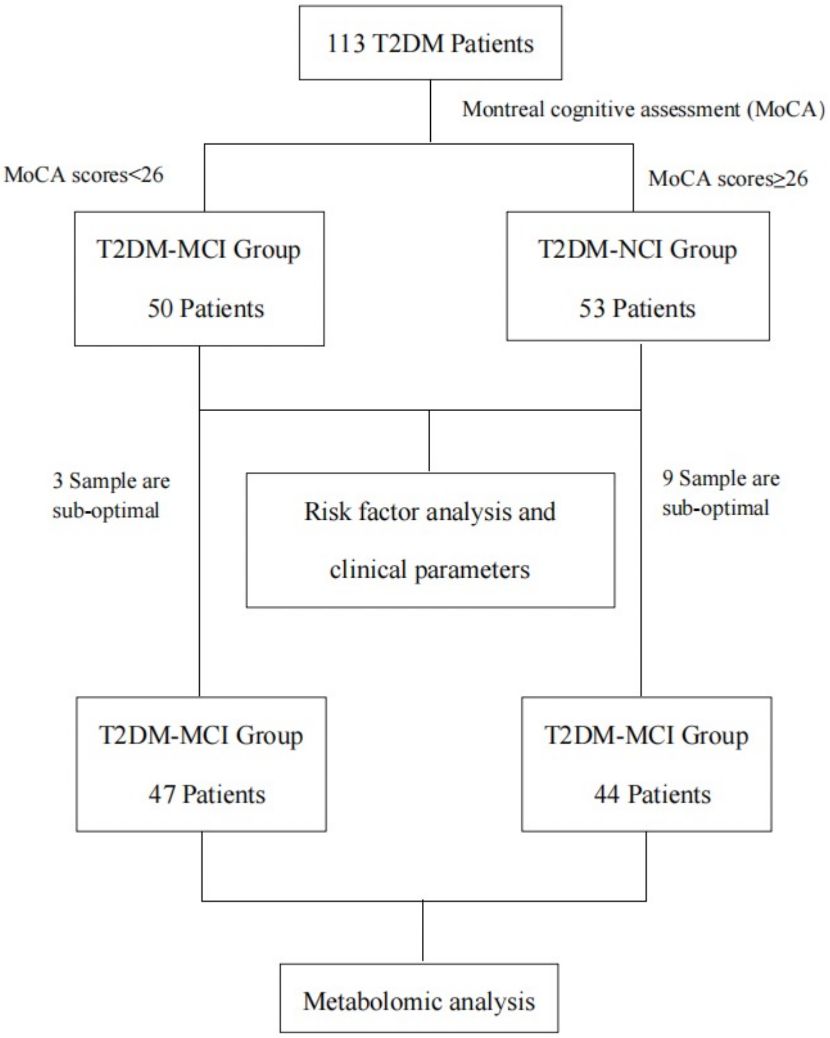

Supplement: Supplementary file 2 [file Image1.pdf]
